# Possible psychosocial strategies for controlling violence against women

**DOI:** 10.4103/0972-6748.62275

**Published:** 2009

**Authors:** Sapna Kumari, Richa Priyamvada, S. Chaudhury, A. R. Singh, A. N. Verma, J. Prakash

**Affiliations:** Department of Psychiatric Social Work, RINPAS, India; 1 Department of Clinical Psychology, RINPAS, India; 2 Department of Psychiatry, RINPAS, India

**Keywords:** Violence, Women, Prevention, Advocacy

## Abstract

Women, the fair sex, are considered to be the weaker sex and one of the most powerless and marginalized sections of our society. Violence against women and girls continues to be a global epidemic. It is present in every country, cutting across boundaries of culture, class, education, income, ethnicity and age. A growing body of research studies indicates that 20% to 50% (varying from country to country) of women have experienced domestic violence. A multilayered strategy that addresses the structural causes of violence against women is needed. Strategies and interventions attempting to address violence against women should be guided by 5 underlying principles: Prevention, protection, early intervention, rebuilding the lives of victims/survivors and accountability. When planning interventions, there are a variety of stakeholders who should be borne in mind. Key areas for intervention include encouraging women empowerment; advocacy and awareness raising; education for building a culture of nonviolence; encouraging active participation of women in political system; resource development; direct service provision to victims, survivors and perpetrators; networking and community mobilization; direct intervention to help victims/survivors rebuild their lives; legal reform; monitoring interventions and measures; early identification of ‘at risk’ families, communities, groups and individuals; and data collection and analysis.

Violence against women is any act of gender-based violence that results in, or is likely to result in, physical, sexual or mental harm or suffering to women, including threats of such acts, arbitrary deprivation of liberty, whether occurring in public or private life. It is one of the most pervasive of human rights violations, denying women and girls equality, security, dignity, self-worth and their right to enjoy fundamental freedoms. Violence against women is present in every country, cutting across boundaries of culture, class, education, income, ethnicity and age. The global dimensions of this violence are alarming. Violence against women continues to be a global epidemic that kills, tortures and maims - physically, psychologically, sexually and economically. Everywhere, women are vulnerable to violence and exploitation. Violence against women is a manifestation of historically unequal power relations between men and women, which have led to domination over, and discrimination against, women by men and to the prevention of full advancement of women.

## TYPES OF VIOLENCE

Physical violence includes hitting, slapping, punching, kicking, burning, cutting or otherwise harming the body.

Sexual violence includes rape, assault, forced prostitution, incest, female genital mutilation, sexual harassment, inappropriate/unwanted touching, etc.

Economic violence includes overwork, denial of ownership of property, withholding or taking away earnings, denial of inheritance, withholding education, unequal pay, not being allowed to work, etc.

Emotional violence includes verbal abuse, threats, insults, control, constant criticism, intimidation, humiliation, etc.

## CAUSES OF VIOLENCE

There is no one single factor to account for violence perpetrated against women. Increasingly, research has focused on the interrelatedness of various factors that could improve our understanding of the problem within different cultural contexts.

The important cultural factors are gender-specific socialization, cultural definitions of appropriate sex roles, expectations of roles within relationships, belief in the inherent superiority of males, values that give men proprietary rights over women and girls, notion of the family as a private sphere and under male control, customs of marriage (bride price/dowry) and acceptability of violence as a means to resolve conflict.

The important economic factors responsible for domestic violence are women’s economic dependence on men; limited access to cash and credit; discriminatory laws regarding inheritance, property rights, use of communal lands and maintenance after divorce or widowhood; limited access to employment in formal and informal sectors; and limited access to education and training for women.

The important legal factors are lesser legal status of women, either by written law and/or by practice; laws regarding divorce, child custody, maintenance and inheritance; legal definitions of rape and domestic abuse; low levels of legal literacy among women; and insensitive treatment of women and girls by police and judiciary.

The important political factors are under-representation of women in power, politics, the media and in the legal and medical professions; domestic violence not taken seriously; notions of family being a private sphere and beyond control of the state; risk of challenge to status quo/religious laws; limited organization of women as a political force; and limited participation of women in organized political system.

## CONTRIBUTING FACTORS

Although alcohol and poverty are often identified as causes, they are triggers or contributing factors to violence. They are not the root cause of violence.

## CONSEQUENCES OF VIOLENCE AGAINST WOMEN

The physical consequences are physical injuries - fractures, concussions; poor health - chronic pain, gastrointestinal disorders, permanent disability; and death due to homicide or suicide.

The sexual consequences are unwanted pregnancies; sexually transmitted infections, including HIV; miscarriages; and low birth weight babies.

The emotional consequences are unhappy relationship with partner; emotional distance from, and mistrust by, children; stress, depression, hopelessness, lack of satisfaction, panic disorders, low self-esteem; and drug and/or alcohol abuse.

The economic consequences are loss of economic productivity; fewer hours worked due to injury and illness; and reduction in family and community incomes as a result of costs of treatment.

## CONSEQUENCES OF VIOLENCE AGAINST WOMEN (VAW) FOR CHILDREN

- Children live in fear all the time
- Low self-esteem
- Problems in school, e.g., poor performance
- Violent behavior
- Abnormally sensitive
- Withdrawal from activities
- Sleeping problems

## STRATEGIES AND INTERVENTIONS-AN INTEGRATED APPROACH

A multi-layered strategy that addresses the structural causes of violence against women while providing immediate psychosocial services to victims/survivors ensures sustainability and is the only strategy that has the potential to eliminate this scourge. When planning strategies and interventions, there are a variety of stakeholders who should be borne in mind.

- At the level of the family, the stakeholders include women, men, adolescents and children.
- Within the local community, partnerships have to be developed with traditional elders, religious leaders, community-based groups, neighborhood associations, men’s groups (e.g., village farmers’ associations), local councils and village-level bodies.
- Within civil society, the range of partners includes professional groups, women’s and men’s groups, NGOs, the private sector, the media, academia and trade unions.
- At the state level, strategies must be designed in partnership with the criminal justice system (the police, judiciary and lawyers); the health care system; parliament and provincial legislative bodies; and the education sector.
- At the international level, the stakeholders include international organizations (such as the United Nations agency, the World Bank and the regional development banks).

Key areas of intervention include the following:

- Advocacy and awareness raising in every section of society is the most important need of the hour.
- Education for building a culture of nonviolence. Curricula that teach nonviolence, conflict resolution, human rights and gender issues should be included in elementary and secondary schools, universities, professional colleges and other training settings.
- Training for health providers is necessary for early screening and identification of women who are suffering violence.
- Resource development and utilization also need to be taken into consideration.
- Networking and community mobilization and creating awareness about the impact of violence on communities convey the importance of preventing violence against women and girls.
- Direct intervention and direct service provision to victims/survivors and perpetrators would help them rebuild their lives.
- Legal reform. One step towards upholding the right of women to equal protection under the law is to enact violence legislation that specifically prohibits violence against women.
- Data collection and analysis. Reliable data on the magnitude, consequences and the economic and health costs of gender-based violence will help to place the issue on the policy makers’ radar screen.
- Early identification of ’at risk’ families, communities, groups and individuals would help a lot in preventing violence against women and girls.

Above all, 4 underlying principles should guide all strategies and interventions attempting to addresses violence:

- Prevention
- Protection
- Early intervention
- Rebuilding the lives of victims/survivors

## CONCLUSION

Violence against women and girls is globally one of the most prevalent yet relatively hidden and ignored issues. It is a health-related, legal, economic, educational, developmental and, above all, a human rights issue. There is a need for coordinated and integrated policy responses; implementation of existing legislation; and greater accountability from government; in order to eliminate this violence. In recent years, there has been a greater understanding of the problem of violence, its causes and consequences. Women also have to learn to be assertive and accept new roles for themselves. They have to develop an optimistic and hopeful approach to life. They need to be empowered through education, employment opportunities, legal literacy and right to inheritance. Human rights education and information regarding violence should be provided to them because this is a matter of their absolute rights.
